# Social Mobility, Self‐Selection, and the Persistence of Class Inequality in Electoral Participation

**DOI:** 10.1111/1468-4446.70018

**Published:** 2025-07-29

**Authors:** Giacomo Melli, Nan Dirk de Graaf, Geoffrey Evans

**Affiliations:** ^1^ Department of Sociology University of Oxford Oxford UK; ^2^ Trinity College Oxford UK; ^3^ Nuffield College Oxford UK

**Keywords:** diagonal reference models, electoral participation, intergenerational class mobility, longitudinal data, selection effects, working class non‐voting

## Abstract

In recent decades, non‐voting among the British working class has increased substantially, contributing to widening class‐based inequality in electoral participation. This study examines the impact of occupational class mobility on the intergenerational transmission of electoral participation in two ways. First, by applying Diagonal Reference Models to data from the British Household Panel Survey and the UK Household Longitudinal Study covering eight General Elections. Through this, we estimate the impact of mobility on the relative influence of class of origin and class of destination. Second, by examining patterns of non‐voting during the early years of adulthood in order to estimate the degree to which class patterns of non‐voting among occupationally mature adults reflect processes of prior self‐selection, rather than the pattern of non‐voting associated with occupational class of destination. The findings indicate that upwardly mobile individuals are more likely to vote, but only after they have experienced occupational mobility into the middle class, thus suggesting a process of acculturation into the class of destination that diminishes the influence of their class origins. Conversely, individuals who are downwardly mobile from the middle class are less likely to vote. However, this lower level of participation is already apparent earlier in life, before they experience adult occupational mobility. This suggests a pre‐existing pattern indicative of selection effects. These dynamics, in the context of balanced patterns of upward and downward mobility, reinforce class inequalities in electoral participation and suggest that relative differences in turnout between social classes are likely to remain stable or even widen.

## 1 Introduction

Electoral participation is socially stratified, with individuals from lower socio‐economic backgrounds, young people, and immigrants consistently exhibiting lower levels of political engagement and turnout (Evans and Hepplewhite 2022; Evans and Tilley 2017). Nonetheless, unlike characteristics such as ethnicity, that are fixed from an early age, individuals can and do change their social class. Despite the pivotal role of voting in the democratic process, little is known about the intergenerational transmission of electoral participation across social classes and the impact of social mobility. This study addresses this gap by investigating the relative impact of classes of origin and destination on electoral participation in Britain.

This study examines whether electoral participation is primarily shaped by long‐lasting socialisation effects from the class of origin, whether socially mobile individuals undergo a process of acculturation, defined as political alignment with the behavioural pattern typical of their class of destination (Blau 1956; de Graaf et al. 1995), or whether differences in participation precede mobility, suggesting a process of self‐selection. In other words, we test three potential explanations: enduring influence of class‐based socialisation, acculturation following mobility, or pre‐existing differences among individuals who become socially mobile. Recent research suggests that some degree of self‐selection may occur among the socially mobile (Ares and van Ditmars 2025; van Ditmars 2020), implying that the observed differences in participation cannot be fully ascribed to the experience of mobility itself. Rather, it may be that certain behavioural tendencies were already in place prior to the experience of intergenerational class mobility.

This paper examines these propositions by making use of the potential offered by available panel data. We apply Diagonal Reference Models (DRMs; Sobel 1981, 1985) to analyse the combined British Household Panel Survey (BHPS) and UK Household Longitudinal Study dataset (UKHLS, University of Essex and Institute for Social and Economic Research 2023), covering eight General Elections between 1992 and 2019. DRMs enable us to statistically estimate the impact of mobility on the relative influence of class of origin and class of destination, providing a robust framework to test the dynamics underpinning the relationship between social mobility and electoral participation (Shi and Gugushvili 2025).

We focus on Britain, where class and political behaviour have historically been intertwined (Butler and Stokes 1969; Goldthorpe 1999; A. F. Heath et al. 1985). However, while prior to the 2000s the differences in electoral participation rates across Britain's social classes were relatively moderate, recent evidence indicates a shift towards electoral participation structured along class lines, with particularly high abstention rates among the working class (Evans and Tilley 2017). In a further contrast to the latter half of the 20th century, which was characterised by a period of absolute upward mobility, Britain is now characterised by balanced patterns of intergenerational mobility, with similar proportions of individuals experiencing upward and downward social class mobility (Bukodi et al. 2020). This implies that in the 21st century, the effects of downward mobility are likely to be of greater electoral importance than was previously the case.^1^

Our findings reveal evidence of self‐selection into downward mobility, suggesting that the British working class is becoming increasingly characterised by low participation rates, driven by the sorting process of downwardly mobile non‐voters joining a class with an already low propensity to vote (Evans and Tilley 2017). In contrast, those joining the middle class tend to acculturate to its higher voting propensity, aligning with the middle‐class immobile voters. These findings suggest that underlying differences in electoral participation between classes in Britain are likely to persist or even widen in the future.

The paper will proceed as follows: it begins with an outline of the context of the study, then reviews the relevant literature and presents the hypotheses. This is followed by a detailed description of the research design, the presentation of the empirical results, and a concluding discussion.

## 2 The Context

**FIGURE 1:**
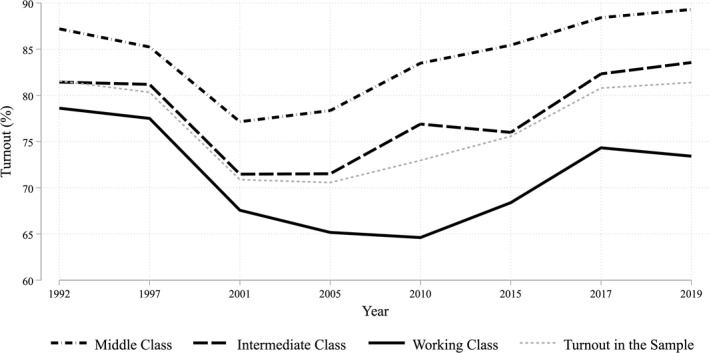
Class turnout in Britain. Data from BHPS/UKHLS (1995–2020). As the question on turnout is retrospective, the graph considers the waves immediately following the general election. The Middle Class consists of managerial, administrative and higher professional occupations and lower professional and higher technical occupations. The Intermediate Class consists of intermediate occupations (clerical and administrative occupations, sales and service occupations, technical and auxiliary occupations), employers in small organisations, and the self‐employed. The Working Class consists of technical and lower supervisory occupations, semi‐routine and routine occupations.

The turnout rate of British General Elections has fluctuated significantly in recent decades and has become more class‐based. From the 1950s to the 1990s, turnout was consistently above 70%, with limited differences between social classes. However, in 2001, it reached the peak of abstention when it dropped to just 59.4%. After that point, it only partially recovered, with the level of abstention consistently between 30% and 35% and returning to 40% in the 2024 election. The class‐based turnout figures calculated from the BHPS/UKHLS panel surveys are shown in Figure 1. These closely mirror those obtained using the British Election Study and British Social Attitudes cross‐section surveys used by Evans and Tilley (2017). They show that following the drop in turnout in the 2001 election, the working class has not recovered to previous levels of participation, with class participation rates diverging most noticeably in the 2005 and 2010 elections when, unlike in the intermediate and middle classes, there was no rebound in participation levels.

Evans and Tilley (2017) argue that this working‐class abstention is attributable to a transformation in the Labour Party's political agenda, rather than a significant alteration in the material conditions of the working class. During its period in office, from 1997 to 2010, the Labour Party targeted its political claims towards the middle class, leaving the working class without clear political representation. More precisely, they show that Labour supporters tend to abstain from voting when they believe that the Labour Party is no longer concerned with the needs of the working class. From 2010 onwards, working class voters also moved to the Scottish National Party (SNP) in Scotland and the United Kingdom Independence Party (UKIP) in England and Wales. But neither party was able to fully compensate for increased rates of relative working‐class abstention.

Evans and Tilley's (2017) analysis, however, does not examine how patterns of social mobility might shape the impact of the political context on class differentials in turnout. In contemporary Britain, nearly equal proportions of individuals experience upward or downward mobility, with approximately one‐third of the population moving in each direction^2^ (Bukodi et al. 2020). This trend, also evident in the Nordic countries and France, is primarily driven by an expansion of the salariat class, which has since abated, resulting in a larger number of individuals raised in the middle class than the number of middle‐class jobs available. This has significant implications for electoral behaviour. A substantial proportion of the working class now comprises individuals socialised in a different class, meaning that those socialised in contexts where voting is the norm, increasingly find themselves in social classes where abstention is widespread. This study thus examines the influence of both class background and current class position on voter turnout in contemporary Britain, where a significant share of the population has experienced downward mobility into a working class with low levels of electoral participation.

## 3 Socialisation, Mobility, and Electoral Participation

Intergenerational social mobility has long been viewed as a significant factor in shaping political behaviour. Durkheim (1897) postulated that mobility, due to its capacity to bring about a radical change in individuals' social networks and expectations, can lead to increased or reduced inclination to participate. Similarly, Sorokin (1959) hypothesised that mobility has detrimental effects on individuals' social integration. Individuals who experience social mobility are engaged in relationships with multiple social groups, each of which has its own set of preferences and expected behaviours (Lindh et al. 2021; Lipset 1960). In particular, long‐distance mobility entails a significant shift along the social ladder and a marked change in social and economic circumstances.

Behavioural research indicates that voting often becomes habitual: individuals who vote in one election are significantly more likely to do so in subsequent ones (Aldrich et al. 2011; Dinas et al. 2024; Gerber et al. 2003; Green and Shachar 2000). But voting is also shaped by structural inequalities (Evans 2000). The resource model of electoral participation holds that individuals with greater resources, such as income, education, and civic skills, are more likely to vote (Verba et al. 1995). In Britain, however, class‐based differences in turnout cannot be explained by resource inequality alone. As Evans and Tilley (2017) argue, declining participation among the working class is better understood as a response to political exclusion and perceived lack of representation. Despite growing concern about declining turnout among working class voters in many countries (Evans and Tilley 2017; O. Heath 2018; Rennwald 2020), the intergenerational inheritability of class differences in electoral participation has not hitherto been explored.

Existing studies on political socialisation have primarily focused on the influence of parents on their children's political preferences (Jennings and Niemi 1968; Neundorf et al. 2013) and party identification (de Graaf et al. 1995; Nieuwbeerta and Wittebrood 1995). This is especially relevant for Britain, where class‐based socialisation is a relatively strong determinant of individuals' future political preferences and identities, irrespective of intergenerational mobility (Ares and van Ditmars 2025; Evans et al. 2022; Helgason and Rehm 2024; Macfarlane 2022). Ares and van Ditmars (2023) employed Linear Probability Models to investigate the influence of parental social class on left/right party support in Germany, Switzerland, and Britain. Their findings indicated that parental social class exerts a significant impact on left/right party support, regardless of current class position. However, they did not find significant differences in Labour party support between upwardly mobile and immobile individuals in Britain. In contrast, Paterson (2008), using DRMs, found that in Britain, individuals who experience upward intergenerational class mobility undergo a process of acculturation, adopting the political attitudes and civic engagement patterns of their destination class rather than retaining those of their class of origin. Jerrim and Kaye (2024) similarly observed that upwardly mobile individuals are significantly more likely to support the Conservative Party than those who remain in their class of origin. Further, Helgason and Rehm (2024), using a fixed effects model with a continuous impact function on BHPS data from 1991 to 2008, demonstrated that intergenerationally upwardly mobile individuals adopt increasingly economically conservative views over time.

There is a notable absence of research investigating the influence of social class background and intergenerational class mobility on adults' electoral participation. Gidengil et al. (2016) employ Finnish register data from 1999 to examine the relationship between parental and young adult (aged 18–30) electoral participation. Bhatti and Hansen (2012) employed Danish register data from 2009 to assess this relationship in an even narrower age group (18–22 years). Yet, neither study examines the class basis of such intergenerational effects on electoral participation. We therefore address this lacuna in knowledge by examining the long‐term socialisation effects of parental social class on the electoral participation of adults, leveraging panel data from eight British general elections.

A central question is whether electoral participation is more strongly shaped by class of origin or class of destination, and what role mobility plays in this process. It is thought that the political preferences and behavioural tendencies of mobile individuals should be situated somewhere between those of immobile individuals in their origin and destination classes (Bartley and Plewis 2007; de Graaf et al. 1995; Weakliem 1992).

The ‘acculturation hypothesis’ posits that mobile individuals tend to adopt the behavioural patterns typical of their class of destination (Blau 1956; de Graaf et al. 1995; de Graaf and Ultee 1990). One implication of this is that the influence of the class of destination on political behaviour is expected to increase with age, as longer exposure fosters greater alignment with the norms and preferences of that class (de Graaf et al. 1995; de Graaf and Ultee 1990). de Graaf et al. (1995) identify this pattern with regard to political preferences in Britain, Germany, the Netherlands, and the USA, while Helgason and Rehm (2024) observed similar findings for economic preferences in Britain. Based on this, we formulate the following hypothesis:

H 1 Acculturation Hypothesis: The relative influence of class of destination on electoral participation will be stronger for older socially mobile individuals than for younger socially mobile individuals.

However, this alignment is an observable outcome that may result from multiple underlying mechanisms, and the specific factors driving individuals to adopt the behavioural patterns of their destination class remain uncertain. It may reflect a process of social learning, whereby individuals adopt behaviours, such as voting, based on the practices of those they now interact with (Blau 1956; de Graaf et al. 1995; Lindh et al. 2021). Alternatively, individuals' decision to vote may be influenced by the degree to which they believe political parties represent their destination class, both nominally and via their issue appeals (Evans and Tilley 2017; Robison et al. 2021; Thau 2019). As these processes may operate differently for upwardly and downwardly mobile individuals, these trajectories are analysed separately.

Given that upward mobility entails a transition from less participatory to more participatory classes (Evans and Hepplewhite 2022), which also receive greater attention from political parties (Evans and Tilley 2017; Robison et al. 2021; Thau 2019), we expect that upwardly mobile individuals will adopt a higher propensity to vote.

H 2 Upward Mobility Hypothesis: Upwardly mobile individuals will have a high propensity to vote, aligning more closely with immobile individuals in their class of destination than with immobile individuals in their class of origin.

The ‘acculturation hypothesis’ may also apply to downward mobility. The behavioural alignment may arise from external cues, such as observing common abstention in the new social environment, and from internal reassessment of available choices. In this view, abstention can be understood as a rational response to political unresponsiveness and disillusionment (Evans and Tilley 2017; Robison et al. 2021; Rosset 2023). Downwardly mobile individuals may thus come to resemble their new class, in which abstention is often a common choice (Evans and Tilley 2017; Rennwald 2020). While disentangling these mechanisms empirically remains difficult, both point to the same outcome: a low level of participation associated with downward mobility.

This is supported by a growing body of empirical evidence on other political outcomes. Gugushvili et al. (2025) show that, in Europe, experiences of downward mobility are associated with support for far‐right parties. In the British context, Jerrim and Kaye (2024) find that individuals experiencing downward mobility are less likely to support the Conservative Party. Similarly, Kurer and van Staalduinen (2022) find that, in Germany, individuals whose current socioeconomic status falls below that of their background are more likely to abstain from voting, even after accounting for other individual socioeconomic factors.

As said, downward mobility may also increase political disillusionment, further dampening electoral participation. Individuals who perceive the political system as unresponsive are less likely to vote (Rosset 2023). In the USA, Newman (1988) argues that individuals experiencing downward mobility are likely to attribute their circumstances to systemic failures. Similarly, in Belgium, downward educational mobility is associated with heightened political dissatisfaction (Daenekindt 2017), while in the Netherlands, it is linked to lower political trust (Daenekindt et al. 2018). These findings suggest that intergenerational downward mobility may erode political engagement, leading us to formulate the following hypothesis:

H 3 Downward Mobility Hypothesis: Downwardly mobile individuals will have a low propensity to vote, aligning more closely with immobile individuals in their class of destination than with immobile individuals in their class of origin.

An alternative explanation for differences in participation among mobile individuals is self‐selection into mobility. This process suggests that changes in political behaviour are not a consequence of the experience of mobility into a different occupational class itself, but rather stem from pre‐existing characteristics shaped before entry into the labour market.

Previous research has identified sorting and selection effects related to class mobility concerning positions on the political ideology spectrum (van Ditmars 2020), as well as economic conservatism and gender attitudes (Ares and van Ditmars 2025). This study investigates potential differences in electoral participation among young individuals socialised in the same class of origin who later diverge in their occupational class.

The factors driving early differences in political behaviour remain uncertain, though family and education are likely key influences. The family represents a key *locus* for the transmission of political behaviours and attitudes, where selection effects may arise from within‐class heterogeneity in parental engagement with politics (Jeannet 2022; Jennings et al. 2009; Kuhn et al. 2021; Langsæther et al. 2022; Neundorf et al. 2015; van Ditmars 2020, 2023). Education also plays a significant role in shaping political preferences, providing another context in which early differentiation in political behaviour can take root (Hastie 2007; Lancee and Sarrasin 2015; Stubager 2008). Together, these early influences can significantly shape electoral participation at the first election, which, in turn, strongly predicts future turnout (Aldrich et al. 2011; Dinas et al. 2024; Gerber et al. 2003; Green and Shachar 2000). The first election, therefore, provides a key opportunity to assess the impact of sorting processes.

If self‐selection is at play, we would anticipate discernible differences in political engagement among ‘young’ individuals who will later experience intergenerational class mobility compared to their non‐mobile peers, that is, individuals with the same class background. Specifically, those who will later achieve upward mobility should already display a greater propensity for political participation at an early age, while those who will go on to experience downward mobility should show lower levels of engagement. Conversely, if such differences are absent, changes in the propensity to vote among mobile individuals are likely driven by their new social contexts rather than by pre‐existing characteristics, as described above. We expect to find evidence of self‐selection into mobility in Britain, such that:

H 4a Upward Self‐Selection Hypothesis: Young individuals who will later experience upward mobility will exhibit a higher propensity to vote compared to their young non‐mobile peers from the same class of origin.

H 4b Downward Self‐Selection Hypothesis: Young individuals who will later experience downward mobility will exhibit a lower propensity to vote compared to their young non‐mobile peers from the same class of origin.

## 4 Research Design

### 4.1 Data and Variables

We employ data from the combined dataset of the BHPS and UKHLS (University of Essex and Institute for Social and Economic Research 2023). The use of such panel data enables us to examine not only intergenerational mobility, but also the possibility of self‐selection. We include only individuals who reside in England, Scotland, and Wales. The sample is composed of more than 14,000 individuals, and almost 42,000 person‐year observations.

The dichotomous dependent variable indicates whether individuals voted in the last General Election. To mitigate the potential retrospective bias, the analysis is restricted to the waves immediately following General Election years. Hence, from BHPS waves 5, 7, 11, 15, and from UKHLS waves 2, 7, 8, 9, 10, 11, 12, were selected. As UKHLS waves are conducted over several months, only observations from individuals interviewed in the 12 months following the election are included in the analysis.

Social class is measured using the six‐class version of the National Statistics Socio‐Economic Classification (NS‐SEC): Higher managerial and professional occupations, Lower managerial and professional occupations, Intermediate occupations, Small Employers and Self‐employed, Lower supervisory, Semi‐Routine and Routine occupations. This classification is based on the Erikson‐Goldthorpe‐Portocarero (EGP) schema (Erikson et al. 1979; Erikson and Goldthorpe 1992), which categorises occupations based on employment relations in the labour market (Evans 1992; Evans and Mills 1998). For individuals who are retired or unemployed, we assign their most recently observed class in the panel. If class data is missing in a given wave, we assume it will be the same as the last reported class until a transition is indicated in a subsequent wave. Social class of origin is measured using the highest class of the parents.

Political behaviour is substantially shaped by social class, with the clearest divides found between the working class and the professional managerial class (A. F. Heath et al. 1991; Evans and Tilley 2017; Langsæther et al. 2022). Accordingly, in examining mobility trajectories, we focus on movements between the middle class, the intermediate class, and the working class. The Middle Class encompasses managerial, administrative, and higher professional occupations, as well as lower professional and higher technical roles. The Intermediate Class includes intermediate occupations, such as clerical, administrative, sales, service, technical, and auxiliary roles, alongside small employers and the self‐employed. The Working Class comprises technical and lower supervisory positions, as well as semi‐routine and routine occupations.

In the models, we include age, gender, and wave as covariates. In Britain, as well as in other developed democracies, voting follows a curvilinear pattern with age, reaching its highest point among middle‐aged individuals (Evans 1993; Smets 2016). Only individuals aged thirty‐five or older are included as it is assumed that they have reached occupational maturity. Historically, women exhibited a voting pattern influenced by their partners if they were in stable relationships (de Graaf and Heath 1992). However, this tendency has diminished in recent years (de Graaf and Heath 2023), and as such, we include gender as a control variable. A conservative choice on controls is justified as we are interested in observing the effects of mobility, avoiding the risk of controlling for post‐treatment characteristics, such as household income. By controlling for survey year, we account for period effects. We include in the analyses only individuals with valid information for all relevant variables.^3^

### 4.2 Methodological Approach

In examining the impact of social mobility, it is important to differentiate between three explanatory factors: the class of origin, the class of destination, and the process of social mobility itself. Linear regression models, which are the conventional methodological approach adopted in social mobility research (e.g., Ares and van Ditmars 2023), fail to compute mobility effects (Hendrickx et al. 1993; Sobel 1981, 1985). Specifically, mobility indicators are linearly dependent on both origin and destination classes, which prevents the simultaneous estimation of all three variables—origin, destination, and mobility—within a single linear model, as this would result in perfect multicollinearity.

In contrast, Diagonal Reference Models (DRMs, Hendrickx et al. 1993; Sobel 1981, 1985), which are non‐linear, enable us to disentangle the effects of origin, destination, and mobility. This is achieved by separating mobile and immobile individuals in the mobility table, estimating the diagonal cells separately, while simultaneously apply a single relative weight parameter to capture the influence of origin and destination in the off‐diagonal cells. This allows for a more accurate assessment of the mobility effect, as mobility is no longer conflated with origin and destination effects. DRMs are widely applied in research on mobility and political preferences (Shi and Gugushvili 2025).

The model assumes that the voting likelihood of individuals who are immobile in their class of origin—that is, those who have not experienced mobility—reflects the structural likelihood of voting for that class. These individuals, ‘born and bred’ in their class (Sorokin 1959), are the most straightforward choice when seeking a reference point to gauge the effect of mobility on turnout. They are located in the diagonal cells of the mobility table.^4^

We implement DRMs in their logistic version in Stata 19.5 (Kaiser 2018). The traditional DRMs are adapted to longitudinal data in two ways, as tested by Jerrim and Kaye (2024). First, each individual observation is considered relevant in the computation of the results, regardless of whether the individual class of destination is stable or not. Each surveyed year, the current social class is observed and influences the propensity to vote, regardless of any subsequent mobility. DRMs estimate the relative influence of origin and destination using a single parameter, p. Assuming that p falls between 0 and 1, the influence of the destination class is defined as 1−p, such that any change in the destination class results in a symmetrical change in the effect of the origin class. In other words, while the origin class remains stable, its influence varies in relation to the destination class observed in each surveyed year (see Equation 1). This is not a concern, as the model can incorporate all these movements, even though we have restricted our sample to individuals over the age of 35 precisely to minimise the occurrence of intragenerational movements. Finally, to account for the correlation among repeated observations for each individual, we cluster standard errors at the individual level.^5^

The propensity to vote of individuals who have experienced intergenerational social mobility is a weighted sum of the propensities to vote of individuals who are immobile in the class of origin and destination. This is displayed in Equation (1), where the probability of turnout of the $k^{th}$ observation of the individual n in the ${ij}^{th}$ cell is expressed as a function of $m_{i}{Origin}_{i}$ and $m_{j}{Destination}_{j}$, the population means of the ${ii}^{th}$ and ${jj}^{th}$ cells of the mobility table, and $\varepsilon_{ijn}$, the error term for observations clustered by individual. The variables measuring the classes of origins and destination must have an equal number of categories, resulting in a squared mobility table. The model allows the inclusion of covariates $X_{ijb}$.

(1) $${Turnout}_{ijnk}=1/\left(1+\exp-\left(p*m_{i}\left({Origin}_{i}\right)+\left(1-p\right)*m_{j}\left({Destination}_{j}\right)+\Sigma_{b}\beta_{b}X_{ijb}+\varepsilon_{ijn}\right)\right)$$

The parameter p ‘indicates the salience of origin status, relative to that of destination status, to determine the phenomenon under investigation’ (Sobel 1981, 896). It is restricted to lie between 0 and 1, and can be interpreted as the relative importance of origin compared to destination. If p>0.5, then origin will be more relevant than destination in shaping the outcome.

We will relax the model in different ways. First, we will allow p to have a different value for each origin or destination class to test whether the different class positions have different weights. Hence, we allow p to vary according to the class of origin or destination, giving separate estimates for each origin or destination class. This model implies that, for a given class of origin/destination, each destination/origin class has the same weight. Second, to account for mobility, we extend the model following what was proposed by de Graaf et al. (1995). We include an additive term to p, a dichotomous variable which will measure different combinations of mobility. We will first test the relevance of any kind of upward and downward mobility. However, as we anticipate that only long‐distance class mobility will affect political behaviour, we will test this model using a variable that measures mobility into the middle class (*IntoMiddle*) and the working class (*IntoWorking*), separately and jointly, as reported in Equation (2). Third, to assess the role of age in shaping the effect of upward and downward mobility, as hypothesised in the acculturation hypothesis (H1), we separate mobile individuals into two groups: those between the ages of 35 and 50 and those who are 50 or older.

(2) $${Turnout}_{ijnk}=1/\left(1+\exp-\left(\left(p+p_{m}{IntoMiddle}_{i}+p_{w}{IntoWorking}_{i}\right)*m_{i}\left({Origin}_{i}\right)+\left(1-p-p_{m}{IntoMiddle}_{i}-p_{w}{IntoWorking}_{i}\right)*m_{j}\left({Destination}_{j}\right)+\Sigma_{b}\beta_{b}X_{ijb}+\varepsilon_{ijn}\right)\right)$$

## 5 Results

As is typical of surveys, our sample is more politically engaged than the general population. Nevertheless, given that our focus is on relative rather than absolute comparisons, the alignment of the trend curves in the sample with those of the population is a reassuring indicator of the quality of the data.

We first explore the data with linear models. The results of the Linear Probability Models of class mobility (Supporting Information S1: Table A5) show a clear trend: individuals who are upwardly mobile are more likely to vote than both those who are immobile and those who are downwardly mobile. Furthermore, the results of the Linear Probability Models of origin and destination (Supporting Information S1: Table A6), as well as the panel Logistic Regression models with random effects (Supporting Information S1: Table A7), indicate that both higher background and current class increase the propensity to vote. There are notable distinctions between Higher and Lower Managers and the rest of the sample.

However, as we argued above, linear models have limitations in their ability to disentangle the effects of origin, mobility, and destination. Even if we were to introduce a statistical interaction between origin and destination, we would only obtain a comparison between the different combinations of classes. DRMs address this issue and provide more theoretically grounded reference points.

The following section will present a series of DRMs and assess their performance using the Bayesian Information Criterion (Raftery 1995), which allows model selection in a predictive context, where parsimony should be favoured. In general, a difference of 10 points or more in BIC score between two models indicates that the model with the lower BIC score performs statistically better than the other model (Raftery 1995, 139).

Table 1 shows a nested model comparison of our DRMs. For the baseline model (i.e., Model 1), we observe an origin weight p of 0.37, which is significantly lower than the destination weight of 0.63 (i.e., 1−p). This indicates that, in the basic model without mobility specifications but controlling for age, gender, wave, and clustering errors within individuals, for mobile individuals class of destination is more important than class of origin for the propensity to vote. In Model 2, p is allowed to vary according to the class of origin. This implies that the influence of the class of origin on the outcome will be the same for all individuals, whereas the weigth for the destination class varies for each origin class. Conversely, in Model 3, p is allowed to vary by class of destination. Model 1 performs statistically better than the other models, and it is also more parsimonious. It is therefore the preferred option for subsequent analyses. Nevertheless, a discernible differentiation has already emerged between those who have moved into the middle class, and particularly into the Higher Managers and Professionals, and the rest of the sample (Supporting Information S1: Table A11).

**TABLE 1:** Logistic diagonal reference models for effects of class of origin and destination and mobility on electoral participation.

Model		p	$p_{x}$	$p+p_{x}$	*BIC*
1	Baseline model $p*m_{i}\left({Origin}_{i}\right)+\left(1-p\right)*m_{j}\left({Destination}_{j}\right)$	0.37*** (0.11)			37290.6
2	By origin $p_{i}*m_{i}\left({Origin}_{i}\right)+\left(1-p_{i}\right)*m_{j}\left({Destination}_{j}\right)$				37321.7
3	By destination $p_{j}*m_{i}\left({Origin}_{i}\right)+\left(1-p_{j}\right)*m_{j}\left({Destination}_{j}\right)$				37312.0
4	Upward mobility into Middle Class $\left(p+p_{m}{IntoMiddle}_{i}\right)*m_{i}\left({Origin}_{i}\right)+\left(1-p-p_{m}{IntoMiddle}_{i}\right)*m_{j}\left({Destination}_{j}\right)$	0.32*** (0.05)	0.12 (0.08)	0.44 (0.13)	37297.8
5	Upward mobility into Middle Class by age $\left(p+p_{a}{IntoMiddle}_{i}*{Age3550}_{i}+p_{b}{IntoMiddle}_{i}*{Age51}_{i}\right)*m_{i}\left({Origin}_{i}\right)+\left(1-p-p_{a}{IntoMiddle}_{i}*{Age3550}_{i}-p_{b}{IntoMiddle}_{i}*{Age51}_{i}\right)*m_{j}\left({Destination}_{j}\right)$	0.32*** (0.05)	*Age 35–50* 0.23** (0.09) *Age 51+* −0.02 (0.10)	0.55 (0.14) 0.30*** (0.15)	37296.3
6	Downward mobility into Working Classes $\left(p+p_{m}{IntoWorking}_{i}\right)*m_{i}\left({Origin}_{i}\right)+\left(1-p-p_{m}{IntoWorking}_{i}\right)*m_{j}\left({Destination}_{j}\right)$	0.45*** (0.04)	−0.25** (0.08)	0.20*** (0.12)	37285.1
7	Downward mobility into Working Classes by age $\left(p+p_{d}{IntoWorking}_{i}*{Age3550}_{i}+p_{e}{IntoWorking}_{i}*{Age51}_{i}\right)*m_{i}\left({Origin}_{i}\right)+\left(1-p-p_{d}{IntoLower}_{i}*{Age3550}_{i}-p_{e}{IntoWorking}_{i}*{Age51}_{i}\right)*m_{i})*m_{j}\left({Destination}_{j}\right)$	0.45*** (0.04)	*Age 35–50* −0.22* (0.09) *Age 51+* −0.27** (0.10)	0.23*** (0.13) 0.18*** (0.14)	37295.5
8	Mobility into Middle and Working Classes by age $\left(p+p_{a}{IntoMiddle}_{i}*{Age3550}_{i}+p_{b}{IntoMiddle}_{i}*{Age51}_{i}+p_{d}{IntoWorking}_{i}*{Age3550}_{i}+p_{e}{IntoWorking}_{i}*{Age51}_{i}\right)*m_{i}\left({Origin}_{i}\right)+\left(1-p-p_{a}{IntoMiddle}_{i}*{Age3550}_{i}-p_{b}{IntoMiddle}_{i}*{Age51}_{i}-p_{d}{IntoLower}_{i}*{Age3550}_{i}-p_{e}{IntoWorking}_{i}*{Age51}_{i}\right)*m_{j}\left({Destination}_{j}\right)$	0.45 (0.07)	*IntoMiddle* *Age 35–50* 0.11 (0.10) *Age 51+* −0.15 (0.10) *IntoWorking* *Age 35–50* −0.25* (0.10) *Age 51+* −0.27 (0.12)	0.56 (0.17) 0.30*** (0.17) 0.20*** (0.17) 0.18* (0.18)	37304.3

*Note:* BHPS/UKHLS (1995–2020). *AnyDown* takes the value 1 if the observation experienced every type of downward mobility, and 0 if the observation remained immobile or experienced upward mobility. *AnyUp* takes the value 1 if the observation experienced every type of upward mobility, and 0 if the observation remained immobile or experienced downward mobility. Individuals whose class of origin is Self‐Employed are not considered in these mobility effects. *IntoWorking* takes the value 1 if the observation experienced downward mobility into the bottom two classes (parental class is not one of the bottom two classes while destination class is), and 0 otherwise. *IntoMiddle* takes the value 1 if the observation experienced upward mobility into the top two classes (parental class is not middle class while destination class is). *Age*3550 takes the value 1 if the observation is between 35 and 50 years of age, and 0 otherwise. *Age*50 takes the value 1 if the observation is older than 50 years old, and 0 otherwise. BIC Bayesian Information Criterion. Every model controls for age, gender, and wave. Model 1 is Model 1 in Supporting Information S1: Table A8. Model 2 is Model 2 in Supporting Information S1: Table A10. Model 3 is Model 3 in Supporting Information S1: Table A10. Model 4 is Model 1 in Table A9. Model 5 is Model 2 in Supporting Information S1: Table A9. Model 6 is Model 3 in Supporting Information S1: Table A9. Model 7 is Model 4 in Supporting Information S1: Table A9. Model 8 is Model 6 in Supporting Information S1: Table A9. Individual cluster‐robust errors in parentheses.

*** *p* < 0.001.

** *p* < 0.01.

* *p* < 0.05, +*p* < 0.1.

We then model the experience of intergenerational class mobility. We first include in the model as covariates two variables measuring any upward or downward mobility. These variables are coded as 1 if there is any mobility between any of the classes, and 0 otherwise. The aforementioned models are presented in Supporting Information S1: Table A7 and show suboptimal performance. Given the uncertainty surrounding their position within the social hierarchy (Jansen 2019), we then exclude from the mobility variables those individuals who were brought up in the Self‐Employed class.^6^ Building upon previous models, Models 4 to 8 present a framework for modelling theoretically relevant social mobility effects. This includes moving into the middle (Models 4 and 5) and working class (Models 6 and 7).

As shown in Model 4, the relative relevance of the class of origin for upwardly mobile individuals into the middle class is 0.44. This figure is not significantly different from 0.5, indicating that for this group, origin and destination classes are equally relevant in determining the outcome. Nevertheless, a disaggregation of upward mobility by age reveals a pattern of acculturation. For middle‐aged individuals, the relative relevance of origin and destination is equivalent. For older individuals, who are likely to have spent a longer period of time in their new class, destination is relatively more relevant than origin. Over time, the salience of destination on the propensity to vote became more pronounced relative to that of origin, supporting the acculturation hypothesis (H1). This pattern is particularly evident among individuals who have experienced upward mobility within the class of Higher Managers and Professionals (Supporting Information S1: Table A11). This class is the most dissimilar to the rest of the population, exhibiting the higher propensity to vote. Overall, these results support the upward mobility hypothesis (H2), as upwardly mobile individuals have a higher propensity to vote than those who remained immobile in their classes of origin.

Individuals experiencing downward mobility exhibit a different behavioural pattern. As illustrated in Model 6 of Table 1, the class of destination is significantly more influential than the class of origin—with an origin weight of 0.20—in determining the propensity to vote of individuals who are downwardly mobile. There are no notable differences by age, as illustrated in Model 7. Individuals who are experiencing downward mobility tend to align their voting patterns with those of the immobile working‐class members they join, indicating a strong tendency towards acculturation. This lends support to the downward mobility hypothesis (H3).

Table 2 presents the predicted probabilities of turnout across different classes of origin and destination. Individuals who move into the Middle Class (Higher and Lower Managerial and Professional classes) are more likely to vote regardless of their class of origin, closely resembling the voting patterns of their immobile peers in their new class. In constrast, those experiencing downward mobility differ more markedly from those who remain immobile in their origin classes than from those immobile in the destination classes. Individuals who are immobile in the Working Class have the lowest rates of voter turnout.

**TABLE 2:** Predicted probabilities of turnout by classes of origin and destination.

Class of origin	Class of destination					
	Routine occupations	Lower supervisory	Self‐employed	Intermediate occupations	Lower managerial	Higher managerial
Routine occupations	0.72	0.75	0.73	0.80	0.84	0.87
Lower supervisory	0.74	0.77	0.75	0.81	0.85	0.88
Self‐employed	0.73	0.76	0.74	0.80	0.84	0.88
Intermediate occupations	0.75	0.78	0.79	0.85	0.87	0.89
Lower managerial	0.76	0.79	0.81	0.86	0.87	0.89
Higher managerial	0.77	0.80	0.84	0.88	0.89	0.91

*Note:* BHPS/UKHLS (1995–2020). Computed from Model 8 in Table 1. The probabilities refer to a 55‐year‐old man in 2005.

### 5.1 Self‐Selection

To assess whether there are elements of self‐selection into mobility, we leverage the advantages of panel data, building on the ideas of Ares and van Ditmars (2025). Specifically, we compare the propensity to vote^7^ among *young respondents* who share the same class of origin but differ in their *future class* of destination, observed at the age of 35 onwards. For the purposes of this analysis, we focus on first electoral participation, measured when individuals are between 18 and 24 years old.^8^ We sort these individuals by their class of destination as observed between the ages of 35 and 55.^9^ Where multiple observations are present, we consider the highest observed class at the older age.

**FIGURE 2:**
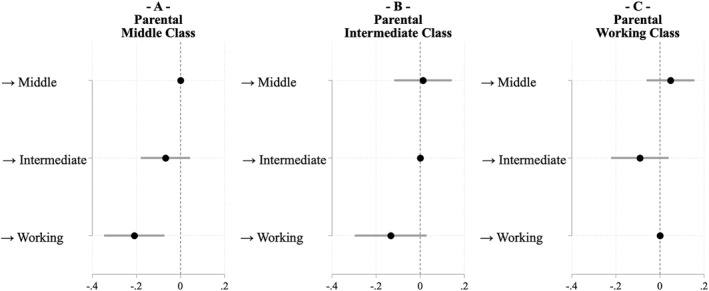
Coefficient Plot of the propensity to vote of individuals between 18 and 24 years old as a function of parental class and future class of destination at age 35 onwards. Computed from Linear Probability Models reported in Table A15 in the Supporting Information S1. BHPS (waves 12–18), UKHLS (waves 2–13).

Because respondents are observed prior to experiencing any social class mobility, this provides a unique opportunity to assess whether sorting processes, that is, a selection effect, rather then the mobility experience itself, shape electoral participation. If self‐selection is not present, we would expect no significant differences in the propensity to vote among young individuals who share primary socialisation, regardless of their eventual future occupational class. As reported in Figure 2, this pattern is observed in the case of *upward* social mobility, whereby individuals raised in the working class who will later become upwardly mobile do not significantly differ in early propensity to vote from those who will remain in the working class (panel C).

Conversely, the data provide evidence of self‐selection in the case of *downward* mobility. Among individuals socialised in the middle class (panel A), those who will later experience downward mobility already exhibit lower levels of political engagement at a young age. Specifically, individuals aged 18–24, who will experience downward mobility have a propensity to vote that is 21 percentage points lower (s.e. 0.07) than their peers who will remain in the higher classes. These results are consistent with the downward self‐selection hypothesis (H4b), but not with upward self‐selection (H4a).

Overall, the findings suggest an asymmetric pattern in how intergenerational class mobility shapes electoral participation. For upwardly mobile individuals, there is evidence of a gradual process of acculturation: over time, their behavioural tendencies increasingly align with the participatory pattern of the middle class, and the influence of their class of origin diminishes. In contrast, the lower propensity to vote observed among downwardly mobile individuals appears to be driven primarily by self‐selection processes. These individuals already exhibited relatively low levels of engagements prior to experiencing downward mobility, suggesting that their long‐term political disengagement reflects pre‐existing tendencies rather than the consequences of mobility itself.

## 6 Conclusion

This paper examined the relationship between intergenerational class mobility and electoral participation, focusing on the intergenerational dynamics of inheritance, acculturation, and self‐selection within the British class structure. To our knowledge, this is the first account of the association between intergenerational social class mobility and electoral participation.

Understanding how class mobility shapes electoral participation helps clarify broader patterns of democratic engagement and exclusion. In recent decades, a marked decline in voter turnout has become a persistent feature of many advanced democracies, including Britain. This decline is not evenly distributed across the electorate. Individuals in less advantaged classes and with lower educational attainment are consistently less likely to vote (Evans and Hepplewhite 2022; Evans and Tilley 2017; Rennwald 2020), raising concerns about the representativeness of democratic institutions.

These developments have occurred alongside a shift in class mobility patterns: in Britain, upward and downward mobility now affect similar proportions of the population (Bukodi et al. 2020). Simultaneously, class has become a stronger predictor of electoral participation, with growing abstention among the working class (Evans and Tilley 2017). Despite these trends, little attention has been paid to how intergenerational class mobility shapes individuals' long‐term patterns of political engagement.

Our findings reveal two key insights. First, individuals who experience upward mobility are more likely to vote compared to those who remain in their class of origin. Importantly, this higher propensity to vote does not appear to be driven by selection into mobility. Rather, it reflects a gradual process of political acculturation: upwardly mobile individuals increasingly align with the participatory patterns typical of their class of destination. These findings underscore the relevance of secondary socialisation processes (de Graaf et al. 1995; de Graaf and Ultee 1990; Helgason and Rehm 2024) and indicate that social mobility can reshape political behaviour in meaningful ways (Langsæther et al. 2022).

Second, individuals who experience downward mobility exhibit consistently lower levels of electoral participation, mirroring the patterns commonly observed among the working class. This lower turnout, however, is not mainly a consequence of mobility. Selection processes play a substantial role: downwardly mobile individuals were already less likely to vote prior to their downward mobility. Their disengagement appears to reflect enduring dispositions, potentially shaped by earlier political socialisation and educational experiences, rather than solely by their current class position. These findings extend recent work on the political consequences of downward mobility (Ares and van Ditmars 2025; Kurer and van Staalduinen 2022), not only by demonstrating selection effects but also by examining its effects on electoral participation.

Those who remain immobile within the working class display the lowest levels of electoral participation. Unlike the upwardly mobile, they have not been exposed to more participatory class environments, nor have they been socialised in a middle‐class *milieu*. As individuals ‘born and bred’ (de Graaf et al. 1995; Sorokin 1959) in a class that has received diminishing attention from Labour since the 1990s, they often perceive themselves as politically unrepresented (Evans and Tilley 2017). This long‐standing sense of exclusion is reflected in a stable pattern of abstention.

Working class abstention is not limited to Britain. Cross‐national evidence shows that working‐class abstention has become increasingly common in other advanced democracies, albeit to varying degrees (Rennwald 2020). In many European countries—including France, the Nordic states, and Britain—rates of downward mobility are now on par with those of upward mobility. In Southern and Eastern Europe, downward mobility affects up to 30%–40% of the population (Bukodi et al. 2020). As a result, working‐class electorates are increasingly composed of downwardly mobile individuals who are politically disengaged. This development raises important concerns about the long‐term legitimacy of democratic institutions. Future comparative research should further unpack these dynamics and their implications for political participation across different contexts. Further work examining the shaping of self‐selection itself, with particular attention to how family background and education influence early patterns of political engagement, is needed.

The findings have significant implications for the political context in Britain. The middle class is likely to become increasingly defined by high levels of political participation, as it both loses non‐voters who become downwardly mobile and politically mobilises the upwardly mobile. In contrast, the working class will remain characterised by low turnout, further reinforced by the arrival of more downwardly mobile non‐voters. This dual process will intensify class‐based inequalities in participation, in response to which vote‐seeking political parties may be even more strongly motivated to appeal to middle class interests. Given the growing share of Britons experiencing downward mobility, and assuming no significant changes on the political supply side, existing class‐based gaps in turnout and representation are thus likely to widen further.

## Conflicts of Interest

The authors declare no conflicts of interest.

## Supporting information

Supporting Information S1

## Data Availability

The data are accessible upon registration and approval of the appropriate licence at http://doi.org/10.5255/UKDA‐SN‐6614‐19.

## Endnotes

^1^
We examine social mobility with respect to social class rather than income, as the former is more capable of capturing the advantages and disadvantages of a social position and their intergenerational transmission (Erikson and Goldthorpe 2010; Goldthorpe 2013).

^2^
As indicated by Bukodi et al. (2020), this figure refers to any movement between any class in the five‐class version of the ESeC scheme (Rose and Harrison 2007).

^3^
Descriptive statistics can be found in Supporting Information S1: Table A1.

^4^
Reported in Supporting Information S1: Table A4.

^5^
Although not explicitly modelled, intragenerational mobility is taken into account. While the analysis of the effect of changing class during the working career is beyond the scope of this paper, our approach is flexible, as it also accounts for the possibility of intragenerational class movements. Although, given that we restrict our analysis to individuals over the age of thirty‐five, the resulting intragenerational mobility rates are limited. See Supporting Information S1: Table A3 in the Supplementary Materials for further details.

^6^
As the hierarchical position of the Self‐Employed is ambiguous and its composition is heterogeneous (Jansen 2019), it is challenging to ascertain which movements are genuinely upward or downward. We therefore exclude this class from the analysis.

^7^
The analyses were replicated using vote intention, with the results presented in Supporting Information S1: Tables A16–A18.

^8^
Analyses based on narrower age groups are presented in Supporting Information S1: Table A19. Despite the smaller sample sizes, the results are consistent with those of the main analysis.

^9^
Further information and descriptive tables can be found in the Supplementary Materials, in Supporting Information S1: Tables A12–A14.
