# Supplementary material for: Social Mobility, Self‐Selection, and the Persistence of Class Inequality in Electoral Participation
Source: Br J Sociol. 2025 Jul 29;76(5):1040–51. doi: 10.1111/1468-4446.70018 (PMC12668251; doi:10.1111/1468-4446.70018)
Supplement: Supplementary file 1 — Supporting Information S1 [file BJOS-76-1040-s001.pdf]

Social Mobility, Self-Selection, and the Persistence of Class Inequality in Electoral Participation.

Supplementary Materials

Figure A1. Class Turnout in Britain. 2

Table A1. Descriptive Statistics. 3

Table A2. Sample Numerosity by Classes of Origin and Destination. 4

Table A3. Intragenerational Mobility in the Sample. 5

Table A4. Intergenerational Social Mobility in Britain (%). 7

Table A5. Results of Linear Probability Models of Class Mobility and Electoral Participation. 8

Table A6. Results of Linear Probability Regression Models with Random Effects of Electoral Participation. 9

Table A7. Results of Panel Logistic Regression of Electoral Participation as a Function of Classes of Origin and Destination. 10

Table A8. Diagonal Reference Models for Any Type of Upward or Downward Social Mobility. 11

Table A9. Diagonal Reference Models with Upward Mobility into Middle Class and Downward Mobility into Working Class. 12

Table A10. Diagonal Reference Models with *p* by Origin and Destination. 13

Table A11. Diagonal Reference Models with Upward Mobility into Higher Managers and Professionals Class and Downward Mobility into Working Class. 15

Table A12. Self-Selection Analysis of Electoral Participation. Mobility Table of Table A14. 16

Table A13. Self-Selection Analysis of Electoral Behaviour. Proportion of Individuals who will achieve higher education by Class of Origin and Destination. 17

Table A14. Average Propensity to Vote of Individuals Between 18 and 24 years old as a Function of their Class of Origin and Future Class of Destination at Age 35 or Older. 18

Table A15. Results of Linear Probability Models on the Propensity to Vote of Individuals Between 18 and 24 years old as a Function of their Class of Origin and Future Class of Destination at Age 35 or Older. 19

Table A16. Average Vote Intention of Individuals Between 18 and 24 years old as a Function of Their Class of Origin and Future Class of Destination at Age 35 or Older. 20

Table A17. Mobility Table of Table A16. 21

Table A18. Self-Selection Analysis of Vote Intention. Proportion of Individuals who will achieve higher education by Class of Origin and Destination, Table A16. 22

Table A19. Results of Linear Probability Models on the Propensity to Vote of Young Individuals as a Function of their Class of Origin and Future Class of Destination at Age 35 or Older. Different Age Groups. 23

Figure A1. Class Turnout in Britain.

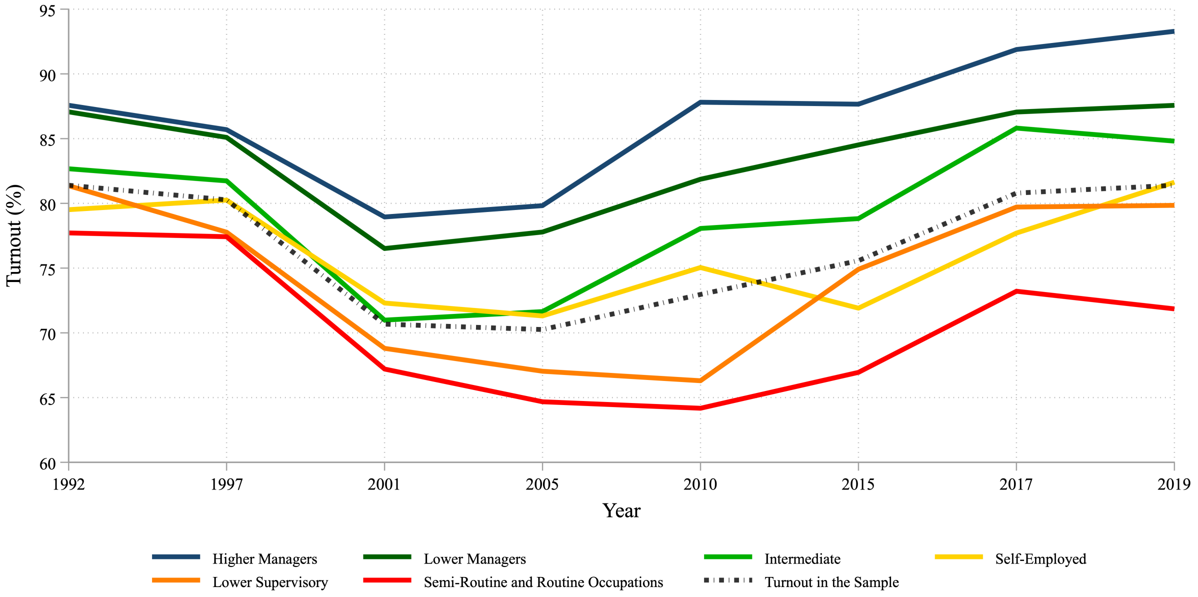

Note: BHPS/UKHLS (1995-2020). As the question on turnout is retrospective, the graph considers the waves immediately following the General Election.

Table A1. Descriptive Statistics.

	Mean/ Percent	Std. Dev
Turnout	0.85	0 .36
Respondent's Social Class		
Higher managerial, administrative, and professional occupation	9.56	
Lower professional and managerial, and higher supervisory	23.49	
Intermediate occupations	14.93	
Small employers and own account workers	10.93	
Lower supervisory and technical occupations	9.75	
Semi-routine and routine occupations	31.34	
Parental Social Class		
Higher managerial, administrative, and professional occupation	5.21	
Lower professional and managerial, and higher supervisory	19.59	
Intermediate occupations	9.66	
Small employers and own account workers	13.15	
Lower supervisory and technical occupations	20.64	
Semi-routine and routine occupations	31.76	
Mobility – direction		
Downward	25.39	
Immobile	39.13	
Upward	35.48	
Education		
Degree	12.18	
Other higher degree	8.89	
A-level etc.	17.49	
GCSE etc.	22.52	
Other qualification	11.29	
No qualification	27.61	
Missing	0.01	
Female	.52	.50
Age	56.04	14.03

Note: BHPS/UKHLS (1995-2020), weighted.

Table A2. Sample Numerosity by Classes of Origin and Destination.

Class of Origin	*Class of Destination*: Routine occupations	Lower supervisory	Self-employed	Intermediate occupations	Lower Managerial	Higher Managerial	Total
Routine occupations	6,250	1,623	1,286	1,791	2,319	590	13,859
	45.10	11.71	9.28	12.92	16.73	4.26	100.00
Lower supervisory	2,699	843	728	1,077	1,775	580	7,702
	35.04	10.95	9.45	13.98	23.05	7.53	100.00
Self-employed	1,725	471	1,133	835	1,446	534	6,144
	28.08	7.67	18.44	13.59	23.54	8.69	100.00
Intermediate occupations	861	324	370	721	1,101	486	3,863
	22.29	8.39	9.58	18.66	28.50	12.58	100.00
Lower Managerial	1,49	484	709	1,229	2,801	1,24	7,953
	18.74	6.09	8.91	15.45	35.22	15.59	100.00
Higher Managerial	288	88	214	353	818	577	2,338
	12.32	3.76	9.15	15.10	34.99	24.68	100.00
Total	13,313	3,833	4,440	6,006	10,260	4,007	41,859
	31.80	9.16	10.61	14.35	24.51	9.57	100.00

Note: BHPS/UKHLS (1995-2020), not weighted. The units of analysis are the individuals' observations in the panel. First row has frequencies, and second row has row percentages.

Table A3. Intragenerational Mobility in the Sample.

Table A3.1. Tabulation.

	Overall		*Between*		*Within*
	Freq.	Percent	Freq.	Percent	Percent
Routine occupations	13313	31.80	5625	39.87	84.80
Lower supervisory	3833	9.16	1930	13.68	71.99
Self-employed	4440	10.61	1929	13.67	77.24
Intermediate occupations	6006	14.35	2600	18.43	73.38
Lower Managerial	10260	24.51	4375	31.01	75.73
Higher Managerial	4007	9.57	1745	12.37	71.03
Total	41859	100.00	18204	129.01	77.51

Note: BHPS/UKHLS (1995-2020), not weighted. The 'Overall' columns summarise results in term of individual panel observations. The 'Between' columns count individuals ever member of a class. The 'Within' column is a measure of stability of class in the sample. N=14110

Table A3.2. Transitions Probabilities of Intragenerational Mobility.

	Class of Destination						
Class of Origin	Routine occupations	Lower supervisory	Self-employed	Intermediate occupations	Lower Managerial	Higher Managerial	Total
Routine occupations	7,475	342	162	222	248	32	8,481
	88.14	4.03	1.91	2.62	2.92	0.38	100.00
Lower supervisory	358	1,836	60	52	144	51	2,501
	14.31	73.41	2.40	2.08	5.76	2.04	100.00
Self-employed	157	43	2,451	45	126	53	2,875
	5.46	1.50	85.25	1.57	4.38	1.84	100.00
Intermediate occupations	252	37	69	3,27	413	85	4,126
	6.11	0.90	1.67	79.25	10.01	2.06	100.00
Lower Managerial	307	100	187	393	5,597	397	6,981
	4.40	1.43	2.68	5.63	80.17	5.69	100.00
Higher Managerial	49	30	77	88	406	2,135	2,785
	1.76	1.08	2.76	3.16	14.58	76.66	100.00
Total	8,598	2,388	3,006	4,07	6,934	2,753	27,749
	30.98	8.61	10.83	14.67	24.99	9.92	100.00

Note: BHPS/UKHLS (1995-2020), not weighted. Rows represent the starting points, and columns represent ending points. First row present frequencies, second row present row percentages.

Table A4. Intergenerational Social Mobility in Britain (%).

Class of Origin	*Class of Destination*<br>Semi-routine and routine occupations	Lower supervisory	Small employers and self-employed	Intermediate occupations	Lower managerial	Higher managerial	
Semi-routine and routine occupations	44.56	12.05	10.04	12.61	16.28	4.46	100.00
Lower supervisory	36.25	11.16	10.30	14.32	20.64	7.32	100.00
Small employers and self-employed	26.08	10.19	18.08	16.03	20.72	8.90	100.00
Intermediate occupations	22.33	8.62	10.60	18.61	27.60	12.23	100.00
Lower managerial	17.98	6.51	8.89	16.64	33.81	16.18	100.00
Higher managerial	11.42	3.37	9.14	15.41	39.37	21.29	100.00
Total	31.34	9.75	10.93	14.93	23.49	9.56	100.00

Note: BHPS/UKHLS (1995-2020). The units of analysis are the individual observations in the panel.

Table A5. Results of Linear Probability Models of Class Mobility and Electoral Participation.

VARIABLES	(1) Any Kind of Mobility	(2) Mobility into Middle and Working Classes	(3) Mobility into Middle and Working Classes by Age
Any Mobility (ref. Immobile)			
Downward	0.01		
	(0.00)		
Upward	0.03***		
	(0.00)		
Downward Mobility into Working Class		-0.03***	
		(0.01)	
Upward Mobility into Middle Class		0.05***	
		(0.00)	
Downward Mobility into Working Class by Age			
35-50			-0.04***
			(0.01)
51+			-0.01
			(0.01)
Upward Mobility into Middle Class by Age			
35-50			0.03***
			(0.01)
51+			0.06***
			(0.01)
Female	-0.01*	-0.01*	-0.01*
	(0.00)	(0.00)	(0.00)
Age	0.00***	0.00***	0.00***
	(0.00)	(0.00)	(0.00)
Wave (ref. BHPS Wave 5 – 1995)			
BHPS Wave 7 – 1997	-0.01+	-0.01+	-0.01+
	(0.01)	(0.01)	(0.01)
BHPS Wave 11 – 2001	-0.10***	-0.10***	-0.10***
	(0.01)	(0.01)	(0.01)
BHPS Wave 15 – 2005	-0.11***	-0.11***	-0.11***
	(0.01)	(0.01)	(0.01)
UKHLS Wave 2/3 – 2010/11	-0.09***	-0.09***	-0.09***
	(0.01)	(0.01)	(0.01)
UKHLS Wave 7/8 – 2015/16	-0.09***	-0.09***	-0.10***
	(0.01)	(0.01)	(0.01)
UKHLS Wave 8/9 – 2017/18	-0.06***	-0.06***	-0.06***
	(0.01)	(0.01)	(0.01)
UKHLS Wave 11/12 – 2020	-0.06***	-0.06***	-0.06***
	(0.01)	(0.01)	(0.01)
Observations	41,859	41,859	41,859

Note: BHPS/UKHLS (1995-2020). Individual cluster-robust errors in parentheses. *** p<0.001, ** p<0.01, * p<0.05, + p<0.1.

Table A6. Results of Linear Probability Regression Models with Random Effects of Electoral Participation.

VARIABLES	(1)<br>Model 1	(2)<br>Model 2
Respondent's Social Class (ref. Intermediate)		
Middle Class	0.04***	
	(0.01)	
Working Class	-0.03***	
	(0.01)	
Parental Social Class (ref. Intermediate)		
Middle Class	0.03***	
	(0.01)	
Working Class	-0.03***	
	(0.01)	
Respondent's Social Class (ref. Higher managerial)		
Lower managerial		-0.01
		(0.01)
Intermediate occupations		-0.03**
		(0.01)
Small employers and own account workers		-0.06***
		(0.01)
Lower supervisory and technical occupations		-0.06***
		(0.01)
Semi-routine and routine occupations		-0.07***
		(0.01)
Parental Social Class (ref. Higher managerial)		
Lower managerial		-0.04**
		(0.01)
Intermediate occupations		-0.04**
		(0.01)
Small employers and own account workers		-0.06***
		(0.01)
Lower supervisory and technical occupations		-0.07***
		(0.01)
Semi-routine and routine occupations		-0.09***
		(0.01)
Female	-0.01	-0.01
	(0.01)	(0.01)
Age	0.00***	0.00***
	(0.00)	(0.00)
Wave (ref. BHPS Wave 5 – 1995)		
BHPS Wave 7 – 1997	-0.01*	-0.01*
	(0.00)	(0.00)
BHPS Wave 11 – 2001	-0.09***	-0.09***
	(0.01)	(0.01)
BHPS Wave 15 – 2005	-0.11***	-0.11***
	(0.01)	(0.01)
UKHLS Wave 2/3 – 2010/11	-0.10***	-0.10***
	(0.01)	(0.01)
UKHLS Wave 7/8 – 2015/16	-0.11***	-0.11***
	(0.01)	(0.01)
UKHLS Wave 8/9 – 2017/18	-0.08***	-0.08***
	(0.01)	(0.01)
UKHLS Wave 11/12 – 2020	-0.09***	-0.09***
	(0.01)	(0.01)
Observations	41,859	41,859
Number of individuals	14,110	14,110

Note: BHPS/UKHLS (1995-2020). Median theta 0.4038. Individual cluster-robust errors in parentheses. *** p<0.001, ** p<0.01, * p<0.05, + p<0.1.

Table A7. Results of Panel Logistic Regression of Electoral Participation as a Function of Classes of Origin and Destination.

VARIABLES	(1) Model 1
Respondent's Social Class (ref. Intermediate)	
Middle Class	0.47***
	(0.07)
Working Class	-0.33***
	(0.07)
Parental Social Class (ref. Intermediate)	
Middle Class	0.33***
	(0.09)
Working Class	-0.28***
	(0.07)
Female	-0.08
	(0.06)
Age	0.05***
	(0.00)
Wave (ref. BHPS Wave 5 – 1995)	
BHPS Wave 7 – 1997	-0.18*
	(0.08)
BHPS Wave 11 – 2001	-1.16***
	(0.08)
BHPS Wave 15 – 2005	-1.34***
	(0.08)
UKHLS Wave 2/3 – 2010/11	-1.27***
	(0.09)
UKHLS Wave 7/8 – 2015/16	-1.34***
	(0.10)
UKHLS Wave 8/9 – 2017/18	-0.99***
	(0.11)
UKHLS Wave 11/12 – 2020	-1.06***
	(0.10)
Observations	41,859
Number of individuals	14,110

Note: BHPS/UKHLS (1995-2020). Individual cluster-robust errors in parentheses. *** p<0.001, ** p<0.01, * p<0.05, + p<0.1.

Table A8. Diagonal Reference Models for Any Type of Upward or Downward Social Mobility.

	(1) DRM	(2) Mobility	(3) Downward	(4) Downward	(5) Upward	(6) Upward
**Diagonal Coefficients**						
Higher managerial	0.75***	0.76***	0.76***	0.80***	0.75***	0.76***
	(0.08)	(0.08)	(0.08)	(0.09)	(0.08)	(0.08)
Lower managerial	0.41***	0.41***	0.41***	0.42***	0.41***	0.41***
	(0.05)	(0.05)	(0.05)	(0.06)	(0.05)	(0.05)
Intermediate occupations	0.15*	0.16*	0.16*	0.16*	0.16*	0.16*
	(0.06)	(0.06)	(0.06)	(0.07)	(0.06)	(0.07)
Self-employed	-0.41***	-0.43***	-0.42***	-0.42***	-0.42***	-0.42***
	(0.06)	(0.07)	(0.06)	(0.06)	(0.06)	(0.06)
Lower supervisory	-0.32***	-0.30***	-0.31***	-0.36***	-0.30***	-0.32***
	(0.06)	(0.06)	(0.06)	(0.07)	(0.06)	(0.07)
Routine occupations	-0.59***	-0.60***	-0.59***	-0.61***	-0.60***	-0.59***
	(0.04)	(0.05)	(0.05)	(0.04)	(0.04)	(0.04)
Origin weight (p)	0.37***	0.36***	0.39***	0.45***	0.34***	0.35***
	(0.04)	(0.06)	(0.05)	(0.06)	(0.05)	(0.05)
Destination weight (1-p)	0.63***	0.64***	0.61***	0.55***	0.66***	0.65***
	(0.04)	(0.06)	(0.05)	(0.06)	(0.05)	(0.05)
Mobility – direction						
Downward		-0.04				
		(0.06)				
Upward		-0.05				
		(0.05)				
Any Downward Mobility			-0.04			
			(0.06)			
p of Downward				-0.18+		
				(0.10)		
Any Upward Mobility					-0.05	
					(0.05)	
p of Upward						0.04
						(0.09)
Age	0.03***	0.03***	0.03***	0.03***	0.03***	0.03***
	(0.00)	(0.00)	(0.00)	(0.00)	(0.00)	(0.00)
Female	-0.04	-0.04	-0.04	-0.04	-0.04	-0.04
	(0.04)	(0.04)	(0.04)	(0.04)	(0.04)	(0.04)
Wave (ref. BHPS Wave 5 – 1995)						
BHPS Wave 7 – 1997	-0.13*	-0.13*	-0.13*	-0.13*	-0.13*	-0.13*
	(0.05)	(0.05)	(0.05)	(0.05)	(0.05)	(0.05)
BHPS Wave 11 – 2001	-0.77***	-0.77***	-0.77***	-0.77***	-0.77***	-0.77***
	(0.05)	(0.05)	(0.05)	(0.05)	(0.05)	(0.05)
BHPS Wave 15 – 2005	-0.86***	-0.86***	-0.86***	-0.86***	-0.86***	-0.86***
	(0.05)	(0.05)	(0.05)	(0.05)	(0.05)	(0.05)
UKHLS Wave 2/3 – 2010/11	-0.82***	-0.82***	-0.82***	-0.82***	-0.82***	-0.82***
	(0.06)	(0.06)	(0.06)	(0.06)	(0.06)	(0.06)
UKHLS Wave 7/8 – 2015/16	-0.87***	-0.87***	-0.87***	-0.87***	-0.87***	-0.87***
	(0.07)	(0.07)	(0.07)	(0.07)	(0.07)	(0.07)
UKHLS Wave 8/9 – 2017/18	-0.64***	-0.64***	-0.64***	-0.64***	-0.64***	-0.64***
	(0.07)	(0.07)	(0.07)	(0.07)	(0.07)	(0.07)
UKHLS Wave 11/12 – 2020	-0.63***	-0.63***	-0.63***	-0.63***	-0.63***	-0.63***
	(0.07)	(0.07)	(0.07)	(0.07)	(0.07)	(0.07)
Constant	0.58***	0.62***	0.60***	0.63***	0.60***	0.59***
	(0.09)	(0.09)	(0.09)	(0.09)	(0.09)	(0.09)
N	41,859	41,859	41,859	41,859	41,859	41,859
AIC	37152.3	37153.6	37153.7	37148.1	37152.1	37153.8
BIC	37290.6	37309.1	37300.7	37295.0	37299.1	37300.7

Note: BHPS/UKHLS (1995-2020). Individual cluster-robust errors in parentheses. *** p<0.001, ** p<0.01, * p<0.05, + p<0.1. BIC Bayesian Information Criterion. AIC Akaike Information Criterion.

Table A9. Diagonal Reference Models with Upward Mobility into Middle Class and Downward Mobility into Working Class.

	(1) Into Middle Class	(2) Into Middle Class by Age	(3) Into Working Class	(4) Into Working Class by age	(5) Combined Mobility	(6) Combined Mobility by age	(7) Combined Mobility by age
Diagonal Coefficients							
Higher managerial	0.79***	0.81***	0.79***	0.79***	0.79***	0.81***	0.81***
	(0.08)	(0.08)	(0.09)	(0.09)	(0.09)	(0.09)	(0.09)
Lower managerial	0.44***	0.43***	0.42***	0.42***	0.42***	0.40***	0.40***
	(0.06)	(0.06)	(0.06)	(0.06)	(0.06)	(0.06)	(0.06)
Intermediate occupations	0.12^{+}	0.12^{+}	0.17*	0.17*	0.17*	0.17*	0.17*
	(0.06)	(0.06)	(0.07)	(0.07)	(0.08)	(0.08)	(0.08)
Self-employed	-0.43***	-0.43***	-0.45***	-0.45***	-0.45***	-0.46***	-0.46***
	(0.06)	(0.06)	(0.06)	(0.06)	(0.07)	(0.06)	(0.06)
Lower supervisory	-0.33***	-0.33***	-0.34***	-0.34***	-0.34***	-0.34***	-0.34***
	(0.06)	(0.06)	(0.06)	(0.06)	(0.06)	(0.06)	(0.06)
Routine occupations	-0.59***	-0.58***	-0.59***	-0.59***	-0.59***	-0.59***	-0.59***
	(0.04)	(0.04)	(0.04)	(0.04)	(0.04)	(0.04)	(0.04)
Origin weight (p)	0.32***	0.32***	0.45***	0.45***	0.45***	0.45***	0.45***
	(0.05)	(0.05)	(0.04)	(0.04)	(0.07)	(0.07)	(0.07)
Destination weight (q)	0.68***	0.68***	0.55***	0.55***	0.55***	0.55***	0.55***
	(0.05)	(0.05)	(0.04)	(0.04)	(0.07)	(0.07)	(0.07)
p of Mobility into Middle Class	0.12				-0.01		
	(0.08)				(0.09)		
p of Mobility into Middle Class by age							
p of between 35 and 50		0.23**				0.11	0.11
		(0.09)				(0.10)	(0.10)
p of older than 51		-0.02				-0.15	-0.15
		(0.10)				(0.10)	(0.10)
p of Mobility into Working Class			-0.25**		-0.25**		-0.26**
			(0.08)		(0.09)		(0.09)
p of Mobility into Working Class by age							
p of between 35 and 50				-0.22*		-0.25*	
				(0.09)		(0.10)	
p of older than 51				-0.27**		-0.27*	
				(0.10)		(0.12)	
Age	0.03***	0.03***	0.03***	0.03***	0.03***	0.03***	0.03***
	(0.00)	(0.00)	(0.00)	(0.00)	(0.00)	(0.00)	(0.00)
Female	-0.04	-0.04	-0.04	-0.04	-0.04	-0.04	-0.04
	(0.04)	(0.04)	(0.04)	(0.04)	(0.04)	(0.04)	(0.04)
Wave (ref. BHPS Wave 5 – 1995)							
BHPS Wave 7 – 1997	-0.13*	-0.13*	-0.13*	-0.13*	-0.13*	-0.12*	-0.12*
	(0.05)	(0.05)	(0.05)	(0.05)	(0.05)	(0.05)	(0.05)
BHPS Wave 11 – 2001	-0.77***	-0.77***	-0.77***	-0.77***	-0.77***	-0.77***	-0.77***
	(0.05)	(0.05)	(0.05)	(0.05)	(0.05)	(0.05)	(0.05)
BHPS Wave 15 – 2005	-0.86***	-0.86***	-0.86***	-0.86***	-0.86***	-0.86***	-0.86***
	(0.05)	(0.05)	(0.05)	(0.05)	(0.05)	(0.05)	(0.05)
UKHLS Wave 2/3 – 2010/11	-0.82***	-0.82***	-0.82***	-0.82***	-0.82***	-0.82***	-0.82***
	(0.06)	(0.06)	(0.06)	(0.06)	(0.06)	(0.06)	(0.06)
UKHLS Wave 7/8 – 2015/16	-0.87***	-0.87***	-0.86***	-0.86***	-0.86***	-0.86***	-0.86***
	(0.07)	(0.07)	(0.07)	(0.07)	(0.07)	(0.07)	(0.07)
UKHLS Wave 8/9 – 2017/18	-0.64***	-0.64***	-0.64***	-0.63***	-0.64***	-0.64***	-0.64***
	(0.07)	(0.07)	(0.07)	(0.07)	(0.07)	(0.07)	(0.07)
UKHLS Wave 11/12 – 2020	-0.63***	-0.63***	-0.62***	-0.62***	-0.62***	-0.63***	-0.63***
	(0.07)	(0.07)	(0.07)	(0.07)	(0.07)	(0.07)	(0.07)
Constant	0.60***	0.67***	0.62***	0.61***	0.62***	0.68***	0.69***
	(0.09)	(0.09)	(0.09)	(0.09)	(0.09)	(0.09)	(0.09)
N	41,859	41,859	41,859	41,859	41,859	41,859	41,859
AIC	37150.9	37140.7	37138.2	37139.9	37140.2	37131.4	37129.5
BIC	37297.8	37296.3	37285.1	37295.5	37295.8	37304.3	37293.7

Note: BHPS/UKHLS (1995-2020). Individual cluster-robust errors in parentheses. *** p<0.001, ** p<0.01, * p<0.05, + p<0.1. BIC Bayesian Information Criterion. AIC Akaike Information Criterion.

Table A10. Diagonal Reference Models with *p* by Origin and Destination.

	(1)	(2)	(3)	(4)	(5)	(6)	(7)
**Diagonal Coefficients**							
Higher managerial	0.75***	0.77***	1.13***	0.84***	0.77***	0.65***	0.80***
	(0.08)	(0.08)	(0.15)	(0.15)	(0.08)	(0.11)	(0.09)
Lower managerial	0.41***	0.43***	0.31***	0.47***	0.43***	0.28*	0.42***
	(0.05)	(0.06)	(0.07)	(0.08)	(0.06)	(0.14)	(0.06)
Intermediate occupations	0.15*	0.14*	0.08	0.13+	0.15*	0.24*	0.16*
	(0.06)	(0.07)	(0.08)	(0.07)	(0.06)	(0.09)	(0.08)
Self-employed	-0.41***	-0.39***	-0.50***	-0.46***	-0.41***	-0.34***	-0.45***
	(0.06)	(0.06)	(0.08)	(0.07)	(0.06)	(0.08)	(0.07)
Lower supervisory	-0.32***	-0.32***	-0.40***	-0.35***	-0.33***	-0.28**	-0.36***
	(0.06)	(0.06)	(0.06)	(0.10)	(0.06)	(0.11)	(0.06)
Routine occupations	-0.59***	-0.63***	-0.62***	-0.62***	-0.60***	-0.55***	-0.57***
	(0.04)	(0.05)	(0.05)	(0.09)	(0.04)	(0.10)	(0.04)
Origin weight (p)	0.37***			0.42***	0.40***	0.42***	0.43***
	(0.04)			(0.08)	(0.08)	(0.08)	(0.08)
Destination weight (1-p)	0.63***			0.58***	0.60***	0.58***	0.57***
	(0.04)			(0.08)	(0.08)	(0.08)	(0.08)
**p by Origin**							
Higher managerial		0.63***					
		(0.12)					
Lower managerial		0.76***					
		(0.07)					
Intermediate occupations		0.77***					
		(0.11)					
Self-employed		0.48***					
		(0.10)					
Lower supervisory		0.41***					
		(0.09)					
Routine occupations		0.62***					
		(0.06)					
**p by Destination**							
Higher managerial			0.66***				
			(0.06)				
Lower managerial			0.30***				
			(0.07)				
Intermediate occupations			0.31**				
			(0.12)				
Self-employed			0.53***				
			(0.11)				
Lower supervisory			0.14				
			(0.13)				
Routine occupations			0.25**				
			(0.09)				
Origin (reg. Intermediate class)							
From Middle class				-0.13			
				(0.12)			
From Working class				-0.02			
				(0.08)			
p of Origin (ref. Intermediate class)							
p of From Middle class					-0.12		
					(0.10)		
p of From Working class					0.03		
					(0.09)		
Destination (ref. Intermediate class)							
Into Middle class						0.17	
						(0.12)	
Into Working class						0.03	
						(0.12)	
p of Destination (ref. Intermediate class)							
p of Into Middle class							0.01
							(0.10)
p of Into Working class							-0.21+
							(0.11)

Age	0.03***	0.03***	0.03***	0.03***	0.03***	0.03***	0.03***
	(0.00)	(0.00)	(0.00)	(0.00)	(0.00)	(0.00)	(0.00)
Female	-0.04	-0.04	-0.05	-0.04	-0.04	-0.04	-0.04
	(0.04)	(0.04)	(0.04)	(0.04)	(0.04)	(0.04)	(0.04)
Wave (ref. BHPS Wave 5 – 1995)							
BHPS Wave 7 – 1997	-0.13*	-0.13*	-0.13*	-0.13*	-0.13*	-0.13*	-0.13*
	(0.05)	(0.05)	(0.05)	(0.05)	(0.05)	(0.05)	(0.05)
BHPS Wave 11 – 2001	-0.77***	-0.77***	-0.77***	-0.77***	-0.77***	-0.77***	-0.77***
	(0.05)	(0.05)	(0.05)	(0.05)	(0.05)	(0.05)	(0.05)
BHPS Wave 15 – 2005	-0.86***	-0.86***	-0.86***	-0.86***	-0.86***	-0.86***	-0.86***
	(0.05)	(0.05)	(0.05)	(0.05)	(0.05)	(0.05)	(0.05)
UKHLS Wave 2/3 – 2010/11	-0.82***	-0.82***	-0.82***	-0.82***	-0.82***	-0.82***	-0.82***
	(0.06)	(0.06)	(0.06)	(0.06)	(0.06)	(0.06)	(0.06)
UKHLS Wave 7/8 – 2015/16	-0.87***	-0.87***	-0.87***	-0.87***	-0.87***	-0.87***	-0.86***
	(0.07)	(0.07)	(0.07)	(0.07)	(0.07)	(0.07)	(0.07)
UKHLS Wave 8/9 – 2017/18	-0.64***	-0.64***	-0.64***	-0.64***	-0.64***	-0.64***	-0.64***
	(0.07)	(0.07)	(0.07)	(0.07)	(0.07)	(0.07)	(0.07)
UKHLS Wave 11/12 – 2020	-0.63***	-0.63***	-0.63***	-0.63***	-0.63***	-0.63***	-0.62***
	(0.07)	(0.07)	(0.07)	(0.07)	(0.07)	(0.07)	(0.07)
Constant	0.58***	0.62***	0.66***	0.63***	0.60***	0.52***	0.62***
	(0.09)	(0.09)	(0.09)	(0.10)	(0.09)	(0.10)	(0.09)
N	41,859	41,859	41,859	41,859	41,859	41,859	41,859
AIC	37152.3	37140.2	37130.6	37153.3	37151.0	37153.3	37145.4
BIC	37290.6	37321.7	37312.0	37308.9	37306.6	37308.9	37300.9

Note: BHPS/UKHLS (1995-2020). Individual cluster-robust errors in parentheses. *** p<0.001, ** p<0.01, * p<0.05, + p<0.1. BIC Bayesian Information Criterion. AIC Akaike Information Criterion. In Models 4 and 5, Middle Class is comprised of Higher Managerial and Lower Managerial classes; Intermediate Class is comprised of Intermediate Occupations, Small Employers and Own Account Workers; Working Class is comprised of Lower Supervisory and Technical Occupations, Semi-Routine and Routine Occupations.

Table A11. Diagonal Reference Models with Upward Mobility into Higher Managers and Professionals Class and Downward Mobility into Working Class.

	(1)	(2)	(3)	(4)
Diagonal Coefficients				
Higher managerial	1.12***	1.08***	1.08***	1.09***
	(0.15)	(0.15)	(0.15)	(0.15)
Lower managerial	0.32***	0.34***	0.34***	0.34***
	(0.06)	(0.06)	(0.06)	(0.06)
Intermediate occupations	0.07	0.10	0.10	0.10
	(0.06)	(0.07)	(0.07)	(0.07)
Self-employed	-0.48***	-0.50***	-0.50***	-0.50***
	(0.06)	(0.07)	(0.07)	(0.07)
Lower supervisory	-0.40***	-0.39***	-0.39***	-0.40***
	(0.07)	(0.06)	(0.06)	(0.06)
Routine occupations	-0.63***	-0.63***	-0.63***	-0.63***
	(0.04)	(0.04)	(0.04)	(0.05)
Origin weight (p)	0.31***	0.38***	0.38***	0.38***
	(0.04)	(0.05)	(0.05)	(0.05)
Destination weight (1-p)	0.69***	0.62***	0.62***	0.62***
	(0.04)	(0.05)	(0.05)	(0.05)
Upward Mobility into Higher Managers and Professionals Class	0.35***	0.29**	0.29**	
	(0.08)	(0.09)	(0.09)	
Downward Mobility into Working Class		-0.18*	-0.18*	
		(0.08)	(0.08)	
Upward Mobility into Higher Managers and Professionals Class by Age				
p of between 35 and 50				0.42***
				(0.09)
p of older than 51				0.11
				(0.11)
p of Mobility into Working Class by Age				
p of between 35 and 50				-0.17*
				(0.09)
p of older than 51				-0.21*
				(0.10)
Age	0.03***	0.03***	0.03***	0.03***
	(0.00)	(0.00)	(0.00)	(0.00)
Female	-0.05	-0.05	-0.05	-0.05
	(0.04)	(0.04)	(0.04)	(0.04)
Wave (ref. BHPS Wave 5 – 1995)				
BHPS Wave 7 – 1997	-0.13*	-0.13*	-0.13*	-0.13*
	(0.05)	(0.05)	(0.05)	(0.05)
BHPS Wave 11 – 2001	-0.77***	-0.77***	-0.77***	-0.77***
	(0.05)	(0.05)	(0.05)	(0.05)
BHPS Wave 15 – 2005	-0.86***	-0.86***	-0.86***	-0.86***
	(0.05)	(0.05)	(0.05)	(0.05)
UKHLS Wave 2/3 – 2010/11	-0.82***	-0.82***	-0.82***	-0.82***
	(0.06)	(0.06)	(0.06)	(0.06)
UKHLS Wave 7/8 – 2015/16	-0.87***	-0.86***	-0.86***	-0.86***
	(0.07)	(0.07)	(0.07)	(0.07)
UKHLS Wave 8/9 – 2017/18	-0.64***	-0.64***	-0.64***	-0.64***
	(0.07)	(0.07)	(0.07)	(0.07)
UKHLS Wave 11/12 – 2020	-0.63***	-0.62***	-0.62***	-0.63***
	(0.07)	(0.07)	(0.07)	(0.07)
Constant	0.66***	0.67***	0.67***	0.70***
	(0.09)	(0.09)	(0.09)	(0.09)
N	41,859	41,859	41,859	41,859
AIC	37134.2	37127.0	37127.0	37116.5
BIC	37281.1	37282.5	37282.5	37289.3

Note: BHPS/UKHLS (1995-2020). Individual cluster-robust errors in parentheses. *** p<0.001, ** p<0.01, * p<0.05, + p<0.1. BIC Bayesian Information Criterion. AIC Akaike Information Criterion.

Table A12. Self-Selection Analysis of Electoral Participation. Mobility Table of Table A14.

	Future Class of Destination			
Class of Origin	Working Class	Intermediate	Middle Class	*Total*
Working class	147	90	180	417
Intermediate	63	83	171	317
Middle Class	53	87	317	457
Total	263	260	668	1,191

Note: *Note*: BHPS (waves 12-18), UKHLS (waves 2-13).

Table A13. Self-Selection Analysis of Electoral Behaviour. Proportion of Individuals who will achieve higher education by Class of Origin and Destination.

	Future Class of Destination		
Class of Origin	Working Class	Intermediate	Middle Class
Working class	.14	.19	.46
Intermediate	.10	.12	.50
Middle Class	.11	.35	.72

Note: BHPS (waves 12-18), UKHLS (waves 2-13). N=1,191.

Table A14. Average Propensity to Vote of Individuals Between 18 and 24 years old as a Function of their Class of Origin and Future Class of Destination at Age 35 or Older.

	Future Class of Destination		
Class of Origin	Working Class	Intermediate	Middle Class
Working Class	.56	.47	.61
Intermediate	.44	.58	.59
Middle Class	.49	.63	.70

Note: BHPS (waves 12-18), UKHLS (waves 2-13). N=1,191.

Table A15. Results of Linear Probability Models on the Propensity to Vote of Individuals Between 18 and 24 years old as a Function of their Class of Origin and Future Class of Destination at Age 35 or Older.

VARIABLES	(1) Parental Middle Class	(2) Parental Intermediate Class	(3) Parental Working Class
Future Class of Destination			
Middle Class	*ref*	0.01	0.05
		(0.07)	(0.06)
Intermediate Class	-0.07	*ref*	-0.09
	(0.06)		(0.07)
Working Class	-0.21**	-0.13	*ref*
	(0.07)	(0.08)	
Constant	0.70***	0.58***	0.56***
	(0.03)	(0.05)	(0.04)
Observations	457	317	417

Note: BHPS (waves 12-18), UKHLS (waves 2-13). N=1,191. *** p<0.001, ** p<0.01, * p<0.05, + p<0.1

Table A16. Average Vote Intention of Individuals Between 18 and 24 years old as a Function of Their Class of Origin and Future Class of Destination at Age 35 or Older.

	Future Class of Destination		
Class of Origin	Working Class	Intermediate	Middle Class
Working Class	.77	.65	.78
Intermediate	.74	.66	.79
Middle Class	.72	.88	.87

Note: BHPS (waves 12-18), UKHLS (waves 2-13). N= 1,029.

Table A17. Mobility Table of Table A16.

Class of Origin	*Future Class of Destination* Working Class	Intermediate	Middle Class	*Total*
Working class	137	80	163	380
Intermediate	57	76	141	274
Middle Class	50	69	256	375
Total	244	225	560	1,029

Note: *Note*: BHPS (waves 12-18), UKHLS (waves 2-13).

Table A18. Self-Selection Analysis of Vote Intention. Proportion of Individuals who will achieve higher education by Class of Origin and Destination, Table A16.

	Future Class of Destination		
Class of Origin	Working Class	Intermediate	Middle Class
Working class	.13	.19	.44
Intermediate	.07	.11	.45
Middle Class	.14	.30	.66

Note: BHPS (waves 12-18), UKHLS (waves 2-13). N=1,191.

Table A19. Results of Linear Probability Models on the Propensity to Vote of Young Individuals as a Function of their Class of Origin and Future Class of Destination at Age 35 or Older. Different Age Groups.

	(1)	(2) *18-20*	(3)	(4)	(5) *18-21*	(6)	(7)	(8) *18-22*	(9)	(10)	(11) *18-23*	(12)
Parental Social Class	Middle	Intermediate	Working	Middle	Intermediate	Working	Middle	Intermediate	Working	Middle	Intermediate	Working
Future Class of Destination												
Middle Class	*ref.*	-0.02	0.16	*ref.*	-0.02	0.14+	*ref.*	-0.00	0.12+	*ref.*	-0.01	0.09
		(0.14)	(0.10)		(0.10)	(0.08)		(0.08)	(0.07)		(0.07)	(0.06)
Intermediate Occupations	-0.13	*ref.*	0.09	-0.12	*ref.*	0.04	-0.05	*ref.*	-0.01	-0.05	*ref.*	-0.06
	(0.10)		(0.12)	(0.09)		(0.10)	(0.07)		(0.08)	(0.06)		(0.07)
Working Class	-0.20+	-0.10	*ref.*	-0.25*	-0.12	*ref.*	-0.24**	-0.15	*ref.*	-0.22**	-0.19*	*ref.*
	(0.12)	(0.16)		(0.10)	(0.13)		(0.09)	(0.10)		(0.08)	(0.09)	
Constant	0.63***	0.53***	0.39***	0.65***	0.59***	0.44***	0.67***	0.60***	0.51***	0.69***	0.60***	0.53***
	(0.05)	(0.12)	(0.07)	(0.04)	(0.09)	(0.06)	(0.03)	(0.07)	(0.05)	(0.03)	(0.06)	(0.05)
Observations	161	91	137	221	142	195	300	203	270	375	258	328

Note: BHPS (waves 12-18), UKHLS (waves 2-13). N=1,191. *** p<0.001, ** p<0.01, * p<0.05, + p<0.1
